# Phytochemical Characterization and Antibiofilm Efficacy of *Piper betle* Extract and Essential Oils Against Clinical *Pseudomonas aeruginosa* and *Staphylococcus* spp. from Small Animals

**DOI:** 10.3390/antibiotics15060549

**Published:** 2026-05-29

**Authors:** Wasapon Soonthoranun, Phirabhat Saengsawang, Wongsakorn Wongwatcharamongkhon, Aumaporn Intajorn, Pareeya Udomkusonsri, Natthasit Tansakul, Udomlak Sukatta, Chantima Pruksakorn

**Affiliations:** 1 Department of Microbiology and Immunology, Faculty of Veterinary Medicine, Kasetsart University, 50 Ngamwongwan Rd., Bangkok 10900, Thailand; wasapon.soo@ku.th (W.S.); fvetphsa@ku.ac.th (P.S.); wongsakorn.wongw@ku.th (W.W.); aumaporn.in@ku.th (A.I.); 2 Department of Pharmacology, Faculty of Veterinary Medicine, Kasetsart University, 50 Ngamwongwan Rd., Bangkok 10900, Thailand; fvetpys@ku.ac.th (P.U.); natthasit.t@ku.th (N.T.); 3 Kasetsart Agricultural and Agro-Industrial Product Improvement Institute, Kasetsart University, Bangkok 10900, Thailand; aapuls@ku.ac.th

**Keywords:** antibiofilm, antimicrobial resistance, essential oils, *Piper betle*, *Pseudomonas aeruginosa*, *Staphylococcus*

## Abstract

Background: Bacterial skin infections in companion animals, particularly those involving multidrug-resistant *Pseudomonas aeruginosa* and methicillin-resistant *Staphylococcus*, pose significant therapeutic challenges due to rising resistance and biofilm formation. While *Piper betle* is well-recognized in human medicine, data on its efficacy against veterinary isolates—and the comparative phytochemistry and potency of its extracts versus essential oils—remains scarce. Objective: This study evaluated the antimicrobial and antibiofilm efficacy of an ethanolic *P. betle* leaf extract compared to a panel of representative essential oils—including *P. betle* leaf oil, clove, tea tree, and plai—against 73 feline and canine clinical isolates. Methods: Minimum inhibitory (MIC) and bactericidal (MBC) concentrations were determined via broth microdilution. Biofilm inhibition was assessed using crystal violet staining to determine the minimum biofilm inhibitory concentration (MBIC). Phytochemical profiles were characterized using gas chromatography–mass spectrometry (GC–MS) and liquid chromatography–quadrupole time-of-flight mass spectrometry (LC–MS/MS-QTOF). Results: *P. betle* leaf extract exhibited superior anti-pseudomonad activity (mean MIC: 1.5 mg/mL; MBC: 1.9 mg/mL), demonstrating significantly greater potency than the tested essential oils. Among the essential oils, clove oil was the most effective against *Staphylococcus* strains (mean MIC: 0.8 mg/mL; MBC: 1.2 mg/mL). Despite 74.4% of *P. aeruginosa* and 90.0% of *Staphylococcus* spp. being strong biofilm producers, the *P. betle* extract demonstrated the highest inhibitory potency against *P. aeruginosa* (MBIC: 0.7 mg/mL) and, alongside clove oil, showed superior efficacy against *Staphylococcus* spp. (MBICs: 0.3 and 0.7 mg/mL, respectively). GC–MS analysis identified chavibetol (confirmed via standard spiking) and hydroxychavicol as the primary extract constituents. LC–MS/MS-QTOF profiling further revealed a prominent phenolic profile, including 2,3-dihydroxybenzoic acid and 3,4-dihydroxybenzaldehyde. Comparative analysis suggests that while clove oil efficacy is primarily driven by high eugenol content, the broad-spectrum potency of the *P. betle* extract arises from a complex phenolic richness, specifically the synergistic presence of hydroxychavicol and chavibetol. Conclusions: These findings confirm the robust potential of *P. betle* extract as a promising plant-based antiseptic for managing biofilm-associated infections and mitigating antimicrobial resistance in veterinary medicine.

## 1. Introduction

Bacterial infections are a major contributor to dermatological caseloads in small animal practice. In both dogs and cats, bacterial skin disease is the second most frequent diagnosis, typically manifesting as a secondary complication of underlying primary disorders, such as allergies or ectoparasites [1]. Dysfunction of the skin barrier predisposes animals to these opportunistic infections, which most commonly present as otitis externa and pyoderma [2,3,4]. Recent data underscore this prevalence, with bacterial otitis externa reported in 16–19% of canine cases [5,6] and 4–7% of feline cases [1]. Additionally, pyoderma affects approximately 11% of dogs and 3% of cats [1,5]. These conditions significantly diminish quality of life by causing pruritus, pain, and malodor, while also posing potential zoonotic risks to owners [7]. Specific bacterial taxa dominate the microbial landscapes of these infections. In canine otitis, *Staphylococcus* spp. are the most frequently isolated pathogens, often occurring alongside *Pseudomonas aeruginosa* or species of *Proteus*, *Enterococcus*, *Streptococcus*, and *Corynebacterium* [7,8]. Similarly, *Staphylococcus* spp. remain the predominant isolates in canine pyoderma, accounting for 87.1% of the cases in recent surveys [9]. Within this genus, *Staphylococcus pseudintermedius* is the primary pathogen, followed by *Staphylococcus schleiferi*, which is increasingly being recognized for its role in chronic and recurrent cases [10,11]. Among Gram-negative pathogens, *P. aeruginosa* is the most prevalent opportunistic isolate recovered from infectious otitis and deep pyoderma in both dogs and cats [3,7,8,9]. Its presence is particularly concerning, as it frequently precipitates persistent, chronic infections that are notoriously difficult to resolve [12].

*P. aeruginosa* is characterized by its remarkable capacity to develop multidrug resistance (MDR), which severely complicates clinical management [13,14]. A longitudinal study of canine skin isolates demonstrated a sharp rise in MDR *P. aeruginosa*, from 5% in 2016 to 34% by 2020 [15]. These isolates exhibited high resistance rates to several clinically significant antimicrobials, including chloramphenicol (83%), ceftiofur (79%), amikacin (77%), and gentamicin (74%) [9]. Approximately 79% of *P. aeruginosa* isolates from canine skin in recent surveys have been classified as MDR strains [9]. Compounding its resistance profile, the pathogenicity of *P. aeruginosa* was further enhanced by its ability to form biofilms. This mechanism renders bacteria significantly more refractory to treatment, increasing the minimum inhibitory concentration (MIC) of antimicrobials by up to 100- to 1000-fold compared to planktonic cells [16,17,18]. This phenotype is highly prevalent in veterinary medicine, with biofilm formation reported in 40–95% of canine otitis isolates [17]. Paralleling these Gram-negative challenges, the increasing prevalence of MDR *Staphylococcus* species presents a significant hurdle in veterinary dermatology. Between 2010 and 2020, isolates from canine and feline skin revealed high frequencies of MDR *S. pseudintermedius* (52.8%) and *S. schleiferi* (44.6%) [19]. Of particular concern is the rise of methicillin-resistant *S. pseudintermedius* (MRSP), with prevalence rates ranging from 12.1% to 40.5% across various canine populations [19,20]. These MRSP isolates typically exhibit extensive co-resistance across multiple antimicrobial classes [21], while also posing broader biosafety risks through transmission to healthy contact animals and the domestic environment [5]. The clinical challenge posed by these staphylococci is further compounded by their capacity to form biofilms. This key virulence factor protects bacteria from both host immune responses and antimicrobial therapy [18]. Recent studies have highlighted the high biofilm-forming potential of pet isolates, with *S. aureus* and *S. pseudintermedius* exhibiting production rates of 94.6% and 51.0%, respectively [22]. Notably, within certain MRSP populations, up to 90.5% of the isolates were classified as moderate-to-strong biofilm producers [23]. Collectively, the synergy between MDR and biofilm formation in both *P. aeruginosa* and *Staphylococcus* spp. drives the severity of chronic infections, often necessitating therapeutic drug concentrations that reach toxic thresholds or that require aggressive surgical intervention [14,24,25].

Topical antibacterial therapy is a cornerstone in managing canine and feline pyoderma and otitis, serving as either a localized monotherapy or a synergistic adjunct to systemic treatment [26]. Beyond achieving clinical cure, long-term topical application is vital to prevent relapse and mitigate antimicrobial resistance (AMR) [26,27]. Current protocols rely heavily on standard antiseptics and chelating agents, such as chlorhexidine and Tris-EDTA, to reduce bacterial load and enhance antimicrobial efficacy [17,28,29]. However, the utility of these agents is increasingly hindered by localized irritation, potential systemic complications, and the emergence of isolates with reduced susceptibility [30,31]. Such limitations, coupled with the threat of AMR, have prompted a search for biocompatible alternatives. Plant-derived extracts offer a promising expansion of the antiseptic pharmacopeia, due to their diverse phytochemical profiles and potential for overcoming established resistance mechanisms [32]. These extracts generally carry a lower risk of allergic reactions and are environmentally sustainable [32]. In veterinary dermatology, they provide a strategic means of treating localized infections while reducing the selection pressure [33,34]. This therapeutic potential is mirrored by market trends, with consumer demand for plant-based products increasing by 15–20% over the last five years [35]. Consequently, identifying specific phytochemicals with potent activity against *P. aeruginosa* and *Staphylococcus* species has become a focal point for modern research.

Plant essential oils and extracts are well recognized for their potent antimicrobial properties [36], and are gaining significant traction as therapeutic alternatives or adjuvants to conventional therapy [37]. For instance, cinnamaldehyde from cinnamon, when potentiated with EDTA, effectively inhibits *P. aeruginosa* isolates from canine otitis externa [38]. Similarly, curcumin has been shown to disrupt quorum sensing and virulence factor expression and reduce biofilm formation in several pathogenic bacteria [39]. In vivo models support these findings, with sage leaf essential oil significantly reducing *P. aeruginosa* growth in infected murine skin wounds [40]. Regarding Gram-positive pathogens, essential oils from thyme, oregano, and savory have demonstrated high efficacy in inhibiting the growth of *Staphylococcus* spp. [41]. Among natural alternatives, a variety of plant essential oils, such as those derived from the Lamiaceae family, have shown promise; however, their efficacy specifically against resistant veterinary isolates requires further validation. In parallel, *Piper betle* has emerged as a particularly noteworthy candidate. Traditionally used for infectious conditions, crude *P. betle* extracts have recently demonstrated potent anti-staphylococcal activity against clinical isolates from canine pyoderma, with mean MIC and MBC values of 252.78 mg/L and 443.06 mg/L, respectively [42]. Notably, this extract exhibited comparable efficacy against both MRSP and methicillin-susceptible strains, outperforming standard topical agents, such as azelaic acid and benzoyl peroxide [42]. While these studies have characterized the properties of *P. betle* against Gram-positive pathogens, a significant knowledge gap remains regarding the comparative efficacy of *P. betle* leaf derivatives versus other established essential oils against *P. aeruginosa* of animal origin. Most existing research relies on standard laboratory strains, which may not fully represent the diverse resistance profiles found in isolates from animal patients. Moreover, comparative studies evaluating multiple plant-derived agents—particularly the differential potency of solvent-based extracts versus volatile oils—against the same set of veterinary pathogens are scarce. Crucially, the correlation between complex phytochemical profiles—identified through both gas chromatography–mass spectrometry (GC-MS) and liquid chromatography–quadrupole time-of-flight tandem mass spectrometry (LC-MS/MS-QTOF)—and their respective inhibitory effects on resistant veterinary pathogens remains poorly defined. Addressing these gaps is essential for identifying the most effective therapeutic candidates in veterinary dermatology. Consequently, this study aims to evaluate the antimicrobial and antibiofilm activities of *P. betle* extract, *P. betle* essential oil, and other selected oils. By integrating high-resolution chemical profiling with comparative efficacy testing, this research seeks to provide a more comprehensive, evidence-based therapeutic strategy for managing resistant dermatologic infections in dogs and cats.

## 2. Results

### 2.1. Antibacterial Activity Screening of Essential Oils Using Disk Diffusion and Broth Microdilution

Nine essential oils and a *P. betle* leaf extract were screened for antibacterial activity against *P. aeruginosa* ATCC 27853 and *S. aureus* ATCC 6538; among these, the *P. betle* leaf extract was the most effective against *P. aeruginosa*, producing a 22.5 mm inhibition zone. Six essential oils—cinnamon, clove, plai, tea tree, *P. betle* leaf oil, and lemongrass—showed inhibition zones ranging from 11.2 to 6.5 mm, whereas citronella, galangal, and turmeric oils exhibited no activity. These six oils also demonstrated inhibitory effects against *S. aureus* ATCC 6538. In the *S. aureus* assay, lemongrass oil exhibited the largest inhibition zone (24.7 mm), followed closely by *P. betle* leaf extract (22.8 mm). The remaining oils (tea tree, clove, *P. betle* leaf oil, cinnamon, and plai) produced zones ranging from 18.0 to 13.5 mm. Based on these disk diffusion results, six essential oils that showed visible inhibition zones against both reference strains, along with the *P. betle* leaf extract, were selected for further broth microdilution testing. The complete inhibition zone data are presented in Table 1.

Initial MIC evaluations were performed on 5 *P. aeruginosa* and 4 *Staphylococcus* strains to guide the selection of plant extracts for subsequent testing. *P. betle* leaf extract, as well as clove, tea tree, and plai oils inhibited the growth of *P. aeruginosa* strains, with mean MIC values ranging from 1.2 to 10.4 mg/mL. These four extracts exhibited significantly lower MICs compared to lemongrass oil, *P. betle* leaf oil, and cinnamon oil (all *p* ≤ 0.0018; Supplementary Table S1). Additionally, the selected extracts demonstrated strong growth inhibition against staphylococcal strains, with mean MICs ranging from 0.2 to 4.0 mg/mL. Notably, *P. aeruginosa* generally required higher concentrations—up to 50-fold for oils such as lemongrass and *P. betle* leaf oil—to achieve inhibition compared to *Staphylococcus* strains. Based on these results, *P. betle* leaf extract, clove, tea tree, and plai oils were selected for subsequent antimicrobial efficacy assays.

The MIC values for both reference strains and clinical isolates are summarized in Table 2. Overall, the antimicrobial activities of the selected extracts showed similar trends between reference strains and clinical isolates, although some variability was observed, particularly among *P. aeruginosa* isolates. In general, clinical strains tended to exhibit slightly higher MIC values, indicating reduced susceptibility compared to reference strains. Based on these findings, *P. betle* leaf extract, clove oil, tea tree oil, and plai oil were retained for further investigation due to their relatively low to moderate MIC values across both bacterial groups. To further validate these findings in a more diverse and statistically robust population, the antimicrobial efficacy of the selected extracts was subsequently evaluated against an expanded panel of 43 *P. aeruginosa* and 30 *Staphylococcus* clinical isolates.

### 2.2. Minimum Inhibitory Concentration and Minimum Bactericidal Concentration of Selected Plant Extracts, Piper betle Leaf Extract, Clove Oil, Tea Tree Oil, and Plai Oil Against Clinical Pseudomonas aeruginosa and Staphylococcus Strains

**Figure 1:**
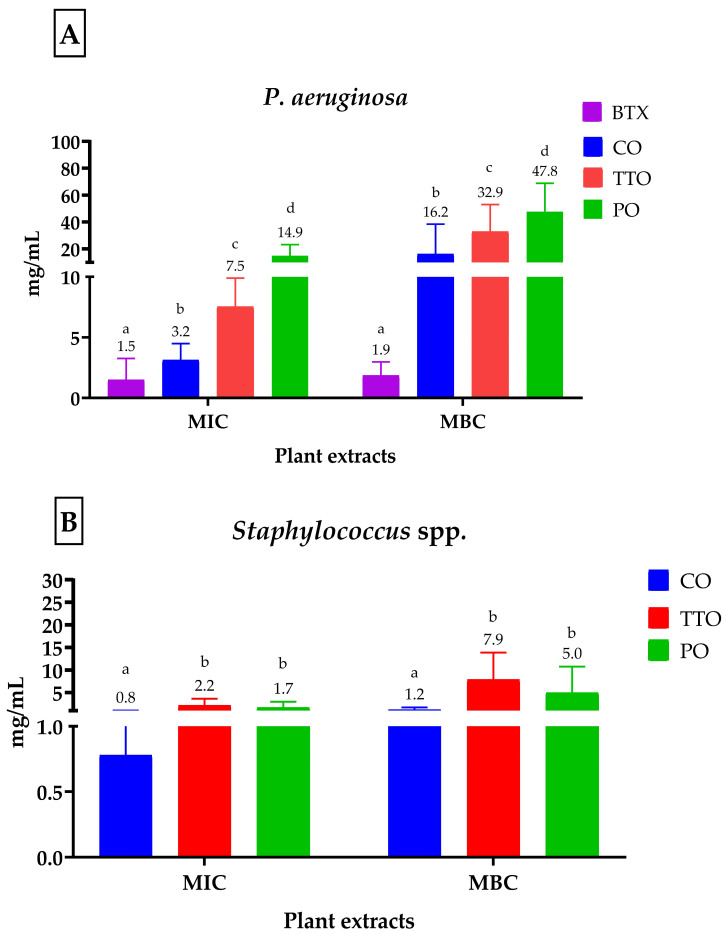
Minimum inhibitory concentrations (MICs) and minimum bactericidal concentrations (MBCs) of plant extracts against clinical strains of (**A**) *Pseudomonas aeruginosa* (*n* = 43) and (**B**) *Staphylococcus* spp. (*n* = 30). BTX, *Piper betle* leaf extract; CO, clove oil; TTO, tea tree oil; PO, plai oil. Significant differences between extract concentrations within the same category (MIC or MBC) are indicated by different letters (a, b, c, d) above the means, as determined by the Kruskal–Wallis One-Way ANOVA (*p* < 0.05). The significance threshold for pairwise comparisons was adjusted to *p* < 0.008 for *P. aeruginosa* and *p* < 0.017 for *Staphylococcus* spp. using the Bonferroni correction.

*P. betle* leaf extract (BTX) exhibited the most potent activity against *P. aeruginosa*, yielding the lowest mean MIC (1.5 mg/mL) and MBC (1.9 mg/mL). This was followed by clove oil (CO; MIC 3.2 mg/mL; MBC 16.2 mg/mL), tea tree oil (TTO; MIC 7.5 mg/mL; MBC 32.9 mg/mL), and plai oil (PO; MIC 14.9 mg/mL; MBC 47.8 mg/mL). Notably, the *P. betle* leaf extract demonstrated bactericidal activity against *P. aeruginosa*, as indicated by an MBC/MIC ratio ≤ 4 [43]. Among the essential oils tested against *Staphylococcus* strains, clove oil demonstrated the highest antibacterial activity, with a mean MIC of 0.8 mg/mL and MBC of 1.2 mg/mL. This was followed by plai oil (MIC 1.7 mg/mL, MBC 5.0 mg/mL) and tea tree oil (MIC 2.2 mg/mL, MBC 7.9 mg/mL). All three oils exhibited bactericidal action against *Staphylococcus* spp. (MBC/MIC ratios ≤ 4) [43]. While *P. betle* leaf extract was highly effective against *P. aeruginosa*, it was not evaluated against *Staphylococcus* strains in this specific assay, as its efficacy against these isolates was previously established [42]. Figure 1 illustrates the comparative MIC and MBC values for all the tested clinical isolates. Detailed pairwise comparisons of the MIC and MBC values for *P. betle* leaf extract, clove oil, tea tree oil, and plai oil against clinical *P. aeruginosa* and *Staphylococcus* strains are provided in Supplementary Table S2. The MIC_50_ and MIC_90_ values were closely aligned for most substances, indicating uniform susceptibility among the tested clinical isolates (Table 3). However, a notable divergence was observed for plai oil against *Staphylococcus* strains, where the MIC_90_ was fourfold higher than the corresponding MIC_50_. Due to its relatively high MIC, plai oil was excluded from subsequent antibiofilm testing against *P. aeruginosa*.

### 2.3. Baseline Multidrug Resistance and Biofilm-Forming Phenotypes of Clinical Isolates

The increasing prevalence of antimicrobial resistance in veterinary dermatology highlights the clinical relevance of this study. A substantial proportion of the clinical *P. aeruginosa* and *Staphylococcus* isolates were resistant to critically important antimicrobial classes, specifically aminoglycosides and methicillin-type drugs. The retained activity of *P. betle* leaf extract against these resistant isolates supports the potential application of multi-target phytochemicals as adjunctive or alternative topical therapies. Among the 43 *P. aeruginosa* isolates, gentamicin resistance and multidrug resistance (MDR) were identified in seven strains. When stratified by host species, the resistance rates for both gentamicin and MDR were comparable between canine and feline isolates, occurring in 17.2% of dogs and 14.2% of cats. Of the 30 canine *Staphylococcus* spp. isolates, MDR was observed exclusively in methicillin-resistant strains; none of the methicillin-susceptible *S. pseudintermedius* (MSSP) isolates exhibited multidrug resistance. Specifically, MDR was detected in 80.0% of methicillin-resistant *S. pseudintermedius* (MRSP) isolates and 70.0% of methicillin-resistant *S. schleiferi* (MRSS) isolates. Furthermore, gentamicin resistance was notably more frequent in MRSP isolates (60.0%) than in MRSS or MSSP isolates (both at 20.0%), as detailed in Table 4.

Given the clinical relevance of biofilm formation in resistant infections, the isolates were further evaluated for their biofilm-forming abilities (Table 5). All 43 *P. aeruginosa* isolates produced biofilms, with 74.4% classified as strong biofilm formers and 25.6% as moderate biofilm formers. An apparent trend between resistance phenotype and biofilm intensity was observed, as a higher proportion of MDR *P. aeruginosa* isolates exhibited strong biofilm-forming ability compared with non-MDR isolates (85.7% vs. 72.2%). Similarly, among the *Staphylococcus* spp. isolates, strong biofilm production was highly prevalent across all groups, ranging from 80.0% to 100.0%, and was observed in all MRSP isolates.

### 2.4. Antibiofilm Activity of Gentamicin Against Clinical Pseudomonas aeruginosa and Staphylococcus Strains

The inhibitory effects of gentamicin on biofilm formation were also tested across the selected isolates. In *P. aeruginosa*, gentamicin-susceptible isolates exhibited lower minimum biofilm inhibitory concentration (MBIC) values (mean 0.0017 mg/mL) than resistant isolates. Notably, gentamicin-resistant *P. aeruginosa* (MIC ≥ 0.0160 mg/mL) showed incomplete biofilm inhibition, even at concentrations reaching 0.0160 mg/mL, with an inhibitory rate of only 49.8%. These findings suggest that gentamicin susceptibility profiles may predict the antibiofilm efficacy of *P. aeruginosa*.

Regarding the *Staphylococcus* strains, gentamicin MBICs were consistently lower in susceptible isolates than in resistant ones. However, a single susceptible *S. pseudintermedius* (MSSP) isolate proved to be an outlier, requiring a high MBIC exceeding 0.0160 mg/mL. Consequently, for both the resistant group and this specific MSSP isolate, biofilm inhibition was only achieved above the 0.0160 mg/mL MIC breakpoint. The classifications of bacterial isolates by gentamicin susceptibility and the corresponding inhibitory concentrations for antibacterial and antibiofilm activities are summarized in Table 6.

### 2.5. Antibiofilm Activity of Piper betle Leaf Extract, Clove Oil, Tea Tree Oil, and Plai Oil on Clinical Pseudomonas aeruginosa and Staphylococcus Strains

Among the plant extracts, *P. betle* leaf extract exhibited the highest antibiofilm efficacy, yielding the lowest mean MBIC against clinical *P. aeruginosa* (0.7 mg/mL). This was followed by clove oil and tea tree oil with mean MBIC values of 4.6 mg/mL and 8.9 mg/mL, respectively, both of which were significantly higher than *P. betle* leaf extract (all *p* < 0.0001; Supplementary Table S3). For *Staphylococcus* spp., *P. betle* leaf extract (0.3 mg/mL) and clove oil (0.7 mg/mL) demonstrated the highest activities and were significantly more potent than tea tree oil (1.7 mg/mL) and plai oil (2.5 mg/mL), both of which exhibited the highest mean MBIC values (all *p* ≤ 0.0014; Supplementary Table S3). Notably, the MBIC of *P. betle* leaf extract against *P. aeruginosa* isolates was lower than the corresponding MIC (Mann–Whitney U test, *p* < 0.0001). No statistically significant differences were observed between the MBIC and MIC for clove and tea tree oils against either species, or plai oil against *Staphylococcus* spp.

**Figure 2:**
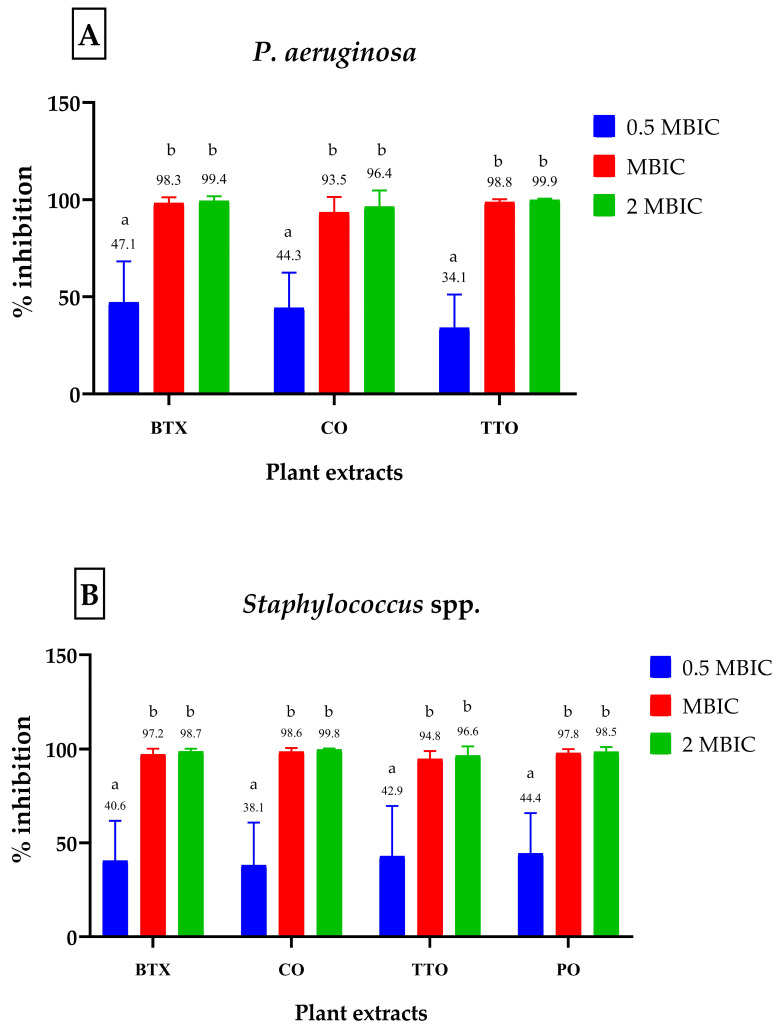
Biofilm inhibitory rate of *Piper betle* leaf extract (BTX), clove oil (CO), tea tree oil (TTO), and plai oil (PO) against clinical strains of (**A**) *Pseudomonas aeruginosa* (*n* = 43) and (**B**) *Staphylococcus* spp. (*n* = 30). Mean values within the same plant extract group carrying different letters (a, b) are significantly different as determined by the Kruskal–Wallis One-Way ANOVA (*p* < 0.05). The significance threshold for pairwise comparisons within each extract-strain group was adjusted to *p* < 0.017 using the Bonferroni correction. MBIC: minimum biofilm inhibitory concentration.

The biofilm-inhibitory potency and dose-dependent effectiveness of *P. betle* leaf extract, clove oil, tea tree oil, and plai oil were evaluated at 0.5 MBIC, MBIC, and 2 MBIC (Figure 2). At 0.5 MBIC, inhibition rates for all extracts remained below 50%. However, *P. betle* leaf extract, clove oil, and tea tree oil each achieved inhibition rates exceeding 90% against *P. aeruginosa* biofilms at both their MBIC and 2 MBIC. These rates were significantly higher than those at 0.5 MBIC (all *p* < 0.0001; Supplementary Table S4). Similarly, all four extracts inhibited *Staphylococcus* spp. biofilms by more than 90% at the MBIC and 2 MBIC levels, showing a significant increase from the 0.5 MBIC (all *p* < 0.0001; Supplementary Table S4).

### 2.6. Gas Chromatography–Mass Spectrometry Profiling of Six Essential Oils and Extracts and Liquid Chromatography–Quadrupole Time-of-Flight Mass Spectrometry Analysis of Piper betle Leaf Extract

GC-MS analysis was employed to characterize the volatile phytochemical profiles of the six plant-derived samples. Compounds were identified via the NIST mass spectral library, with chavibetol specifically confirmed by evaluating its mass shift relative to an eugenol standard. The analysis revealed distinct chemical signatures—expressed as relative abundance (% peak area)—that correlated with the observed antibacterial activities. Clove oil was predominantly composed of the phenylpropanoid eugenol (83.4%) and the sesquiterpene trans-caryophyllene (10.5%). In contrast, tea tree and plai oils were characterized by high relative abundances of monoterpene alcohols, particularly terpinen-4-ol, which accounted for 47.9% and 64.8% of their total volatile compositions, respectively. Tea tree oil also contained *p*-cymene (8.9%), γ-terpinene (8.4%), and α-terpineol (6.3%), whereas plai oil contained sabinene (5.5%), *p*-cymene (4.5%), and α-terpineol (3.7%).

The oils that exhibited selective inhibition against *Staphylococcus*—lemongrass and *P. betle* leaf oil—presented distinct volatile profiles. Lemongrass oil was dominated by the monoterpene aldehyde isomers geranial (48.2%) and neral (33.7%). Conversely, the *P. betle* leaf oil profile was characterized by a complex mixture of phenylpropanoids, including chavibetol (19.8%), eugenol acetate (14.2%), 4-allyl-1,2-diacetoxybenzene (6.2%), and chavicol acetate (3.1%), alongside a substantial sesquiterpene fraction comprising γ-muurolene (10.3%), trans-caryophyllene (7.5%), and δ-cadinene (7.2%).

The *P. betle* leaf extract, which exhibited the most potent broad-spectrum activity against both *Staphylococcus* and *P. aeruginosa*, shared several phenylpropanoids and sesquiterpenes with its essential oil counterpart, notably chavibetol (44.2%), γ-muurolene (5.3%), and δ-cadinene (2.1%). However, the extract was distinguished by a high relative abundance of hydroxychavicol (26.3%), a compound absent from the volatile oil. This difference likely reflects the extraction process, which enables the recovery of more polar phenolic constituents that are not retained during steam distillation. The comprehensive chemical compositions for all six matrices are detailed in Table 7. Further details regarding the comprehensive GC-MS analysis and minor constituents for all six samples can be found in the Supplementary Information (Tables S5–S10) [44].

To further elucidate the polar and non-volatile chemical profile of the *P. betle* leaf extract, LC–MS/MS-QTOF analysis was performed. Compounds were qualitatively identified based on their molecular ion peaks, characteristic fragment ions, and mass-to-charge ratios (*m*/*z*). As summarized in Table 8, the detected metabolites comprised phenolics, phenolic aldehydes, organic acids, sugars, and nucleosides, with phenolic compounds representing the predominant class. The most abundant constituents were 2,3-dihydroxybenzoic acid (20.2%), followed by 3,4-dihydroxybenzaldehyde (15.2%), 3-hydroxybenzyl alcohol (14.9%), and pyrocatechol (14.1%). Other identified compounds included 4-hydroxybenzoic acid, along with primary metabolites such as ornithine, uridine, and trehalose, which were present at lower relative abundances. The high relative abundance of dihydroxy phenolic derivatives likely contributes to the observed antimicrobial synergy of the *P. betle* leaf extract.

Compounds were identified using LC-MS/MS-QTOF, with identification confirmed via the NIST library and Smart Confirmation algorithms based on accurate mass and fragmentation patterns.

## 3. Discussion

*P. betle* leaf ethanolic extract demonstrated the highest efficacy among the tested candidates, characterized by potent broad-spectrum activity and superior inhibition of both bacterial growth and biofilm formation. Notably, the extract exhibited the highest antibacterial and antibiofilm activity against *P. aeruginosa* and outperformed tea tree and plai oils in suppressing *Staphylococcus* spp. biofilms. These findings align with the traditional use of *P. betle* in Thai medicine [45] and support previous reports of its efficacy against diverse bacteria, including *E. coli*, *K. pneumoniae*, and *Streptococcus* [46,47,48,49,50]. Importantly, while previous studies have reported MIC values ranging from 0.51 mg/mL to 4.8 mg/mL for human-derived *P. aeruginosa* isolates [51,52], the present study expands the evidence base to clinical isolates obtained from canine and feline patients. Preliminary research on a small cohort of canine isolates (*n* = 5) initially reported a consistent MIC of 0.625 mg/mL [53]; however, the present study builds upon these findings by evaluating a substantially larger population of 43 veterinary *P. aeruginosa* isolates. The *P. betle* leaf extract demonstrated potent and uniform efficacy, characterized by MIC_50_ and MIC_90_ values of 1 mg/mL and 2 mg/mL, respectively. The proximity of these benchmarks—centered around a mean MIC of 1.5 mg/mL (0.15% *w*/*v*) and a corresponding MBC of 1.9 mg/mL (0.19% *w*/*v*)—underscores a highly consistent antimicrobial profile across diverse clinical strains. This efficacy, further supported by an MBIC of 0.7 mg/mL, highlights the potential of *P. betle* extract as a targeted therapeutic agent in veterinary dermatology. Notably, the low MBC-to-MIC ratio suggests that the extract exerts primarily bactericidal activity. Although bactericidal agents are not invariably clinically superior to bacteriostatic agents, they may offer an important therapeutic advantage in immunocompromised patients, in whom maximal direct antimicrobial activity may facilitate infection clearance.

While the MIC values determined for *P. betle* leaf extract and clove oil were higher than those reported for purified antibiotics such as gentamicin, they remain clinically relevant for topical veterinary applications. Topical formulations, including antiseptic shampoos and otic solutions, are designed to achieve local concentrations that far exceed these MIC thresholds. Furthermore, unlike target site-specific antibiotics, crude botanical extracts exert multi-targeted antimicrobial effects—disrupting membranes, inducing oxidative stress, and inhibiting both quorum sensing and biofilm formation. Therefore, potency comparisons between purified compounds and complex phytochemical mixtures should be interpreted cautiously within the context of topical therapy. A comparison of these active concentrations alongside standard veterinary therapeutics is summarized in Table 9.

Although the MICs demonstrated by the extract were slightly above the 1 mg/mL threshold often cited for crude extracts [55], they remain within a highly effective range for topical antiseptic applications. The therapeutic applicability of plant-derived extracts should be interpreted within the context of topical dermatological therapy rather than systemic antimicrobial therapy. In veterinary practice, medicated formulations such as shampoos and rinses typically contain active ingredients at concentrations of 1–4% *w/v* (10–40 mg/mL). Therefore, the inhibitory concentrations reported here are substantially lower than those readily achievable in clinical formulations, supporting the practical feasibility of these topical applications.

Furthermore, while direct cytotoxicity testing was not performed in the present study, previously published investigations have shown that *P. betle*-derived phenolics exhibit acceptable safety profiles, demonstrating cell viability in keratinocyte and epithelial cell models at concentration ranges overlapping with the present antimicrobial values [56,57,58]. A summary of this published safety data supporting the feasibility of these topical concentrations is provided in Supplementary Table S11. Nevertheless, comprehensive in vivo safety assessments and further pharmacological studies remain necessary. Factors such as prolonged exposure, skin barrier disruption, otic administration, and species-specific sensitivity may influence tolerability, all of which are required to establish optimal dosing regimens for canine and feline dermatological patients.

GC–MS analysis identified chavibetol as the major constituent of the *P. betle* leaf extract. This finding deviates from several previous studies that reported eugenol as the primary compound, suggesting that the chemical profile of *P. betle* can vary significantly according to geographical origin, environmental conditions, and specific cultivation chemotypes [59,60]. While chavibetol is present in both the extract (BTX) and the essential oil (BTO), hydroxychavicol appears to be characteristic of the extract and may contribute to its enhanced antibacterial profile. Hydroxychavicol has been reported to inhibit bacterial cell division and interfere with iron–sulfur proteins in Gram-negative bacteria, thereby inducing cellular instability [61]. Although the specific mechanisms by which hydroxychavicol affects biofilm formation and quorum sensing in *P. aeruginosa* remain unclear, its activity may be enhanced by the extract’s broader antioxidant and anti-inflammatory properties. While GC–MS primarily detects volatile and semi-volatile constituents, LC–MS/MS-QTOF analysis revealed that *P. betle* leaf extract also contains a diverse profile of non-volatile phenolic compounds with reported antimicrobial activities. These include 2,3-dihydroxybenzoic acid, 3,4-dihydroxybenzaldehyde, 3-hydroxybenzyl alcohol, pyrocatechol, and 4-hydroxybenzoic acid. Notably, 2,3-dihydroxybenzoic acid has been reported to function as an iron chelator that competes with the pathogen’s siderophore-mediated iron uptake, which may contribute to the inhibition of biofilm formation, a critical factor in *P. aeruginosa* virulence [62]. Similarly, 4-hydroxybenzoic acid has been suggested to exert antibacterial effects through disruption of cytoplasmic membrane functions and inhibition of key metabolic enzymes, such as ATPase and phosphotransferases, which can interfere with nucleic acid synthesis [63]. In contrast to essential oils, which are chemically restricted to volatile hydrophobic fractions, crude extracts like *P. betle* leaf extract offer a complex matrix of both volatile and non-volatile multi-class compounds. This chemical diversity often leads to enhanced biological activity through multi-targeted synergistic effects [64]. Consequently, these findings support our observation that *P. betle* leaf extract, with its rich phenolic profile, exhibited superior antimicrobial efficacy against *P. aeruginosa* compared to the narrower phytochemical profile of the essential oils.

In comparison, clove oil has long been utilized to treat bacterial infections and inflammatory conditions, such as acne, due to its high concentration of eugenol [65,66,67]. The antibacterial efficacy of eugenol is primarily attributed to its ability to disrupt the cytoplasmic membrane and penetrate the lipopolysaccharide layer, which leads to the leakage of essential intracellular components [68]. Furthermore, eugenol exerts significant antibiofilm activity by reducing bacterial metabolic rate and inhibiting the production of extracellular polysaccharides, which are critical for biofilm stability [69]. While these properties make clove oil an effective antimicrobial agent, particularly against *Staphylococcus* isolates in this study, its activity was more selective than the broader-spectrum potency demonstrated by the *P. betle* leaf extract.

**Figure 3:**
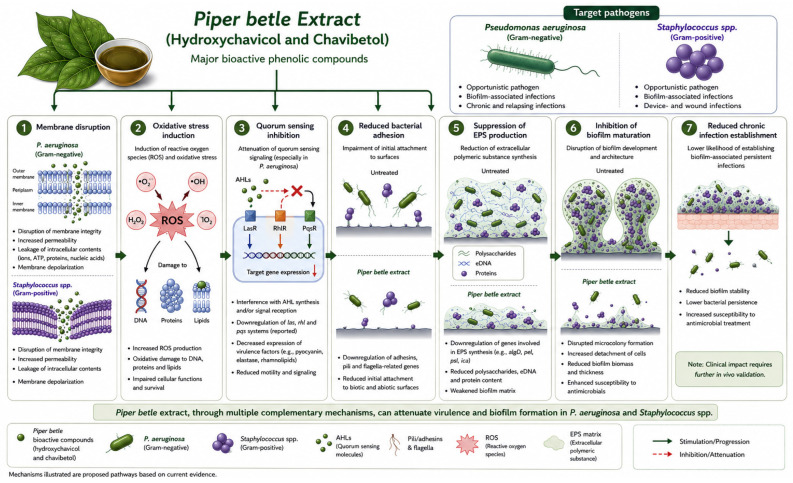
Proposed antibiofilm and anti-virulence mechanisms of *Piper betle* leaf extract against *Pseudomonas aeruginosa* and *Staphylococcus* spp. The extract may disrupt bacterial membrane integrity, induce oxidative stress, attenuate quorum-sensing signaling, reduce bacterial adhesion and extracellular polymeric substance (EPS) production, thereby impairing biofilm maturation and potentially reducing chronic infection establishment. Proposed pathways were summarized based on previous reports on *P. betle* phytochemicals, quorum-sensing attenuation, and biofilm inhibition mechanisms [46,77,78,79,80,81].

Biofilm formation is a key factor in the severity of infections caused by *P. aeruginosa* and *Staphylococcus* spp., as strong biofilm producers often induce more intense inflammation than weaker strains [70,71]. In this study, all canine and feline isolates demonstrated biofilm-forming capabilities, with 74.4% of *P. aeruginosa* and 90% of *Staphylococcus* spp. classified as strong producers. Notably, MDR *P. aeruginosa* isolates tended to exhibit stronger biofilm-forming ability, and all MRSP isolates were classified as strong biofilm producers. These findings further highlight the clinical relevance of biofilm-associated persistence in resistant veterinary infections and support the need for continued investigation of antibiofilm strategies in veterinary medicine. The *P. betle* leaf extract exhibited notable antibiofilm activity against both pathogens, even at sub-inhibitory concentrations, with inhibition rates of 47.1% and 40.6% at 0.5 MBIC for *P. aeruginosa* and *Staphylococcus* spp., respectively. Overall, the extract exhibited the highest inhibitory potency against *P. aeruginosa* and, alongside clove oil, showed superior efficacy against *Staphylococcus* spp., consistent with previous findings [72,73]. Although earlier studies identified 4-chromanol as a key antibiofilm component in *P. betle*, particularly against oral and other Gram-negative bacteria [46,74,75], its exact mechanism of action on the bacterial cell wall requires further investigation. Interestingly, the antibiofilm efficacy of *P. betle* extract appears more pronounced in Gram-negative bacteria, likely because of the distinct properties of their outer membrane barriers [74]. In *P. aeruginosa*, the extract effectively reduced both biofilm formation and production of extracellular polymeric substances [73]. This activity is largely attributed to the disruption of quorum sensing (QS). The extract has been linked to the downregulation of critical QS-related genes (*las*I, *las*R, *las*B, *rhl*I, *rhl*R, and *rhl*A) and reduced expression of quorum-sensing-regulated virulence factors, such as violacein, exopolysaccharides, and pyocyanin [76,77]. The proposed multi-targeted mechanisms underlying these antimicrobial and antibiofilm activities of *P. betle* leaf extract are illustrated in Figure 3.

A particularly noteworthy finding was that the MBIC values of *P. betle* leaf extract against *P. aeruginosa* were lower than the corresponding MIC values. While seemingly counterintuitive, similar sub-inhibitory biofilm disruption has been documented for plant phenolics. Bioactive constituents like hydroxychavicol and chavibetol can impair early biofilm establishment by disrupting quorum sensing, bacterial adhesion, extracellular polymeric substance production, or motility-associated virulence—effectively inhibiting biofilm structure independently of cell death [76,77]. From a clinical perspective, targeting virulence at sub-MICs offers a valuable strategy to prevent chronic infection establishment while avoiding the strong selective pressure that drives antimicrobial resistance during conventional bactericidal therapy.

The antibiofilm activity of *P. betle* leaf extract has been documented against various staphylococci, including *S. aureus* [72] and clinical isolates of *S. pseudintermedius* [82]. In *S. aureus*, the extract is believed to interfere specifically with the initial adherence and subsequent multiplication stages of biofilm development [72], an effect attributed to bioactive constituents, such as phytol, 4-chromanol, and 1,8-cineole [72,74,83]. Building on these findings, this study corroborates the potent activity of *P. betle* extract against a broader range of veterinary clinical isolates, specifically *S. pseudintermedius* and *S. schleiferi*. Regarding *S. pseudintermedius*, previous studies have reported that biofilm inhibition is associated with the downregulation of *ica*A. Molecular interactions between the IcaA protein and specific phenylpropanoids—structurally related to those found in our *P. betle* leaf extract, such as chavibetol and hydroxychavicol—likely drive this inhibitory process, similar to mechanisms reported for eugenol and 4-allyl-1,2-diacetoxybenzene [74]. The significant activity observed against *S. schleiferi* in this study is particularly noteworthy, as it further highlights the broad-spectrum potential of the extract against diverse, veterinary-relevant staphylococci often implicated in chronic skin infections. However, the precise collective mechanism by which these diverse phytochemicals interact to disrupt biofilms across these different species remains to be fully elucidated.

The essential oils of tea tree (*M. alternifolia*) and plai (*Z. cassumunar*) are known for a wide range of biological activities, including antibacterial, antifungal, antioxidant, and anti-inflammatory properties [84,85]. Although previous studies have reported antibacterial and antibiofilm activity against both *S. aureus* and *P. aeruginosa* [86,87,88,89], the efficacy observed in this study was predominantly directed against *Staphylococcus* isolates. The specificity observed in this study may be linked to the distinct chemical profile of the oils. For instance, the composition of plai oil is known to vary significantly based on the cultivation site; dominant constituents have been variously reported as sabinene, terpinen-4-ol, or (E)-1-(3′,4′-dimethoxyphenyl) buta-1,3-diene (DMPBD) [86]. As shown in the chemical profiles of these major constituents, the presence of specific functional groups—such as the hydroxyl group in terpinen-4-ol—is a primary driver of antimicrobial efficacy. In the present study, the phytochemical profile was principally dominated by terpinen-4-ol, aligning closely with findings from Malaysia [90] and explaining the potent staphylococcal inhibition observed, as this compound is recognized as the primary bioactive agent in both tea tree and plai oils [85,87,91,92]. In contrast, the comparatively lower efficacy of these essential oils against *P. aeruginosa*, relative to the *P. betle* leaf extract, may be partly attributed to the absence of non-volatile phenolics and chelators (e.g., 2,3-dihydroxybenzoic acid) present in the crude extract, which may contribute to activity against Gram-negative bacteria.

While *P. betle*, tea tree, clove, and plai are cornerstones of Thai ethnomedicine, their systematic application in veterinary practice remains underdeveloped. The promising antibacterial and antibiofilm results of this study bridge this gap by providing a scientific rationale for their therapeutic use against animal-specific pathogens. One of the primary challenges associated with botanical therapeutics is ensuring batch-to-batch reproducibility, as the composition of crude extracts can vary depending on source material and extraction conditions. To address this potential variability and support translational consistency, chemical fingerprinting using dual GC–MS and LC–MS/MS-QTOF profiling was performed in the present study to characterize the major bioactive constituents of the *P. betle* leaf extract. This approach established preliminary baseline parameters using a single, uniform experimental batch. The extraction yield ranged from 9 to 11%, and relative abundance profiling identified chavibetol (44.2%) and hydroxychavicol (26.3%) as the dominant constituents. These findings provide an initial chemical benchmark to support future batch-to-batch quality control and reproducibility assessments.

For practical clinical use, the *P. betle* extract could be effectively integrated into topical formulations such as medicated shampoos or ear flushes [93]. While its distinctive botanical aroma may influence aesthetic acceptance, it could serve as a natural deterrent to self-licking, thereby ensuring adequate contact time for therapeutic efficacy within the skin microenvironment. Furthermore, potential staining due to the dark pigments of the ethanolic extract can be managed by formulation into wash-off products [94]. Although previous literature notes that the predominant phenolics and antioxidant activity of dried *P. betle* extracts can remain stable for up to six months under light-protected conditions [95], systematic storage stability observations were beyond the scope of this investigation.

Several limitations should be acknowledged when interpreting the findings of this study. First, the antimicrobial and antibiofilm activities were evaluated exclusively under in vitro conditions, which do not fully replicate the complex microenvironment of canine and feline skin or ear canals. Second, no ex vivo or in vivo infection models were employed to assess penetration, persistence, or therapeutic efficacy in biological tissues. Third, direct cytotoxicity and irritation testing were not performed, limiting definitive conclusions regarding clinical safety. Lastly, while qualitative phytochemical components were identified via dual analysis, a precise quantitative standardization of individual bioactive markers was not executed.

To overcome these limitations and build upon the baseline established in this work, future studies should focus on topical formulation optimization, rigorous quantitative phytochemical monitoring of the primary marker compounds (hydroxychavicol and chavibetol) over extended durations, and validation in animal infection models. A crucial avenue for future investigation involves evaluating synergistic interactions between *P. betle* leaf extract and conventional topical antimicrobials or antiseptics, including gentamicin, chlorhexidine, and Tris-EDTA. Because botanical extracts contain multiple bioactive compounds that target distinct bacterial pathways, combination therapy may enhance efficacy while reducing the concentration of conventional agents required—potentially decreasing antimicrobial selective pressure and improving the management of multidrug-resistant, biofilm-associated infections.

Collectively, the findings of this study support the potential utility of *P. betle* leaf extract as a plant-derived topical antiseptic candidate for managing multidrug-resistant and biofilm-associated dermatological infections in companion animals. Although the antimicrobial concentrations observed for the crude extract were higher than those of purified antibiotics, they remain clinically feasible within the context of topical veterinary therapy. Importantly, the pronounced antibiofilm activity observed at sub-inhibitory concentrations suggests that *P. betle* leaf extract may exert anti-virulence effects independent of direct bacterial killing. Combined with a chemically diverse phenolic profile, these findings provide a strong rationale for further translational studies focused on formulation development, safety assessment, and synergistic combination therapy.

## 4. Materials and Methods

### 4.1. Bacterial Isolates

A total of 43 *P. aeruginosa* and 30 *Staphylococcus* spp. clinical isolates were obtained from the culture collection of the Department of Microbiology and Immunology at Kasetsart University. These strains originated from independent clinical cases of canine and feline patients presenting with skin and ear infections at the Kasetsart University Veterinary Teaching Hospital (Bangkok, Thailand), where they were collected during routine diagnostic procedures. As this study used archived bacterial isolates sourced from an existing culture collection and leftover specimens, no new animal sampling was performed. Consequently, no live animals or human participants were involved, and an Institutional Review Board statement was not applicable.

### 4.2. Bacterial Identification and Characterization

The identification of *P. aeruginosa* was confirmed by MALDI-TOF-MS (Vitek MS, bioMérieux, Marcy-l’Étoile, France). The 30 *Staphylococcus* isolates—comprising 10 isolates each of methicillin-resistant *S. pseudintermedius* (MRSP), methicillin-susceptible *S. pseudintermedius* (MSSP), and methicillin-resistant *S. schleiferi* (MRSS)—were characterized by PCR targeting the *nuc* gene for species identification [96] and the *mec*A gene for the detection of methicillin resistance [97]. All isolates were preserved in Luria–Bertani broth with 20% glycerol (MilliporeSigma, Burlington, MA, USA) at −80 °C. Before testing, isolates were subcultured on Trypticase Soy Agar (Becton Dickinson, Franklin Lakes, NJ, USA) for 18–24 h at 37 °C. *P. aeruginosa* ATCC 27853 (Biomedia Thailand Co., Ltd., Bangkok, Thailand) and *S. aureus* ATCC 29213 (DMST 4745) served as standard quality control strains; additionally, ATCC 27853 and *S. aureus* ATCC 6538 (DMST 8013) were utilized as internal references to validate inter-assay reproducibility across different experimental runs. Antimicrobial susceptibility testing (AST) was performed using the VITEK 2 Compact system (Version 08.02) with AST-GN97 cards for *P. aeruginosa* and AST-GP81 cards for *Staphylococcus* spp. (bioMérieux, Marcy-l’Étoile, France). The results were interpreted using the system’s default foundational datasets: Global CLSI 2014 (Vet01-S2) plus Natural Resistance for *P. aeruginosa* and Global CLSI-based plus Natural Resistance 2017 for *Staphylococcus* spp. Multidrug resistance (MDR) was defined as non-susceptibility to at least one agent in three or more antimicrobial categories [98].

### 4.3. Plant Materials and Chemical Composition Analysis

Fresh leaves of *Piper betle* L. (Piperaceae) were sourced from Central Thailand, and a voucher specimen (No. BK086364) was verified and deposited at the Bangkok Herbarium. A crude ethanolic extract (BTX) was subsequently prepared from these leaves according to the method described in a previous study [42]. Essential oils (EOs) of *Cinnamomum zeylanicum* (cinnamon leaf oil: CNO), *Eugenia caryophyllata* (clove flower oil: CO), *Zingiber cassumunar* Roxb (plai rhizome oil: PO), *Melaleuca alternifolia* (tea tree leaf oil: TTO), *P. betle* (*P. betle* leaf oil: BTO), *Cymbopogon citratus* (lemongrass leaf oil: LMO), *Curcuma longa* (turmeric rhizome oil: TMO), *Alpinia galanga* Linn. (galanga rhizome oil: GLO), and *Cymbopogon winterianus* (citronella leaf oil: CTO) were purchased from Thai-China Flavors and Fragrances Industry Co., Ltd. (Bangkok, Thailand). According to the manufacturer’s labeling, all EOs were obtained via steam distillation. The physical properties of the BTX and EOs are documented in Supplementary Table S12, based on the manufacturers’ certificates of analysis and the authors’ direct observations of the BTX extract. The essential oils were used as undiluted reagents for disk diffusion testing. A stock solution of 10× MICs of *P. betle* leaf extract and essential oils of MICs was prepared in 100% DMSO (Merck KGaA, Darmstadt, Germany) and 3% Tween 80 with 1% ethanol (QRëC™, Auckland, New Zealand), respectively. The prepared solutions were filtered using a 0.2 µm syringe filter (Minisart, Sartorius Stedim Biotech, Göttingen, Germany).

The chemical compositions of the selected essential oils (CO, TTO, PO, LMO, BTO) and the ethanolic extract (BTX) were characterized using gas chromatography–mass spectrometry (GC-MS; GC-2030 with a mass-selective detector, Shimadzu, Kyoto, Japan) at the Kasetsart Agricultural and Agro-Industrial Product Improvement Institute (Kasetsart University, Bangkok, Thailand). To ensure optimal resolution of diverse bioactive compounds across different matrices, sample-specific GC-MS protocols—adapted from established methodologies [42,99,100] and summarized in Table 10, were employed. Phytochemicals were identified by comparing their mass spectra with the National Institute of Standards and Technology (NIST) W11N/17M1 library, with relative quantification determined by percentage peak area. For the specific GC-MS analysis of BTO and BTX, a high-purity eugenol standard (100 ppm; MilliporeSigma, Burlington, MA, USA) was spiked into the samples to serve as a reference. The identity of the predominant peak was confirmed as chavibetol through co-chromatography, noting the distinct gas chromatographic retention time and mass spectral fragmentation patterns compared to the added eugenol standard. To characterize the non-volatile constituents within the BTX extract, Liquid Chromatography–Quadrupole Time-of-Flight Mass Spectrometry (LC-MS/MS-QTOF) was performed using a SCIEX ExionLC™ AD Series coupled with an X500R QTOF system (SCIEX, Concord, ON, Canada) at the SciKU Equipment Center (Kasetsart University, Bangkok, Thailand). The analytical parameters for BTX followed the previously described protocols [101]. Compound identification was achieved by comparing the resulting data with the NIST 2017 and Natural Products HR-MS/MS (V2.0) libraries.

### 4.4. Disk Diffusion Method

Nine essential oils were screened for antimicrobial activity against *P. aeruginosa* ATCC 27853 and *S. aureus* ATCC 6538 using the Kirby-Bauer disk diffusion method [102]. Bacterial inocula were prepared and spread onto Mueller–Hinton agar (MHA; Becton Dickinson, Franklin Lakes, NJ, USA). Subsequently, sterile disks (Whatman™ AA DISCS, Cytiva, Little Chalfont, UK) loaded with 10 µL of undiluted essential oil were placed on the inoculated MHA surfaces. Following incubation at 37 °C for 16–18 h, the zones of inhibition were measured. All assays were performed in duplicate, with quality control verified using 10 µg gentamicin disks against the same reference strains.

### 4.5. Determination of Minimum Inhibitory Concentration and Minimum Bactericidal Concentration

Minimum inhibitory concentration (MIC) was determined using the broth microdilution method [42,103]. Briefly, colonies of each isolate were suspended in 0.85% sodium chloride (Vivantis; Shah Alam, Malaysia) and adjusted to a turbidity of 0.5 McFarland standard. The suspension was further diluted to achieve a final concentration of 10^6^ colony-forming units (CFU)/mL. Test reagents were serially diluted twofold in cation-adjusted Mueller–Hinton broth (MHB, Becton Dickinson, Franklin Lakes, NJ, USA), and 100 µL of the working solution was placed in each well of a 96-well polypropylene microplate. Subsequently, 10 µL of prepared inoculum was added. Control wells included MHB with inoculated bacteria (growth control), MHB without bacteria (sterility control), and MHB with test reagents alone to account for background interference (test reagent control). Preliminary testing confirmed that the solvent had no inhibitory effect on bacterial growth. The broth microdilution assay was validated using gentamicin (MilliporeSigma, Burlington, MA, USA) as a quality control, with *P. aeruginosa* ATCC 27853 and *S. aureus* ATCC 29213 as the reference strains. MIC plates were incubated at 37 °C for 18–24 h. Then, 30 µL of 0.02% resazurin dye solution (MilliporeSigma, Burlington, MA, USA) was added to each well, and the wells were incubated for an additional 2–4 h. The lowest concentration of the test substance at which no observable bacterial growth (no change in color) was recorded was the MIC. All MIC assays were conducted in triplicate for each isolate [104]. The minimum bactericidal concentration (MBC) test was performed [105] by inoculating 10 µL aliquots from the MIC test wells onto TSA plates (Becton Dickinson, Franklin Lakes, NJ, USA). After incubation at 37 °C for 18–24 h, colonies were counted, and the MBC was defined as the lowest concentration at which a 99.9% reduction in bacterial viability was observed from the initial inoculum concentration [106].

### 4.6. Biofilm Production and Inhibition Assay

The antibiofilm efficacy of the plant extracts was evaluated following a previously described protocol [107] with minor modifications. Working solutions were prepared via two-fold serial dilution in MHB (Becton Dickinson, Franklin Lakes, NJ, USA) supplemented with 1% glucose. Aliquots (100 µL) of each dilution were dispensed into 96-well microplates (Corning Inc., Corning, NY, USA), followed by inoculation with 10 µL of bacterial suspension (approximately 10^6^ CFU/mL). Three experimental controls included: MHB with 1% glucose (medium control), MHB with 1% glucose and plant extract (extract control), and MHB with 1% glucose with inoculated bacteria (positive control). Following incubation at 37 °C for 24 h, the wells were washed three times with sterile water. Attached cells were fixed with 150 µL of 96% methanol for 15 min, then stained with 200 µL of 0.1% crystal violet for 20 min. After three subsequent washes to remove excess stains, the plates were air-dried at room temperature. Biofilm formation was quantified by solubilizing the crystal violet in 160 µL of 33% glacial acetic acid and measuring the optical density at 590 nm (OD590) using a microplate reader. The minimum biofilm inhibitory concentration (MBIC) was defined as the lowest concentration resulting in no observable biofilm or at least 90% inhibition [108]. Gentamicin was included as a positive antibiofilm control and processed according to the same procedure. *P. aeruginosa* ATCC 27853 served as the quality control reference to ensure inter-assay reproducibility. Biofilm inhibition percentages and production capacities—categorized as none, weak, moderate, or strong—were calculated and classified based on established formulas and OD thresholds [107,109].

$$\%\;Biofilm\;Inhibition=\frac{\left(OD\mathrm{positive}\;\mathrm{control}-OD\mathrm{media}\;\mathrm{control}\right)-(OD\mathrm{experiment}-OD\mathrm{extract}\;\mathrm{control})}{\left(OD\mathrm{positive}\;\mathrm{control}-OD\mathrm{media}\;\mathrm{control}\right)}\times 100$$

### 4.7. Statistical Analysis

Statistical analyses were performed using NCSS software (version 2021; NCSS, LLC, Kaysville, UT, USA). Differences in MIC, MBC, and MBIC across the four extracts were evaluated using the Kruskal–Wallis One-Way ANOVA for both *P. aeruginosa* and *Staphylococcus* spp. This same analysis was used to compare biofilm inhibition rates across different tested concentrations within each extract-strain group. For all analyses, when significant differences were detected, a post hoc analysis using the Bonferroni correction was applied for multiple pairwise comparisons. Differences in MIC values between species, as well as comparisons between MIC and MBIC within the same bacterial group, were analyzed using the Mann–Whitney U test. Statistical significance was defined as *p* < 0.05. All assays were conducted in triplicate, and results are expressed as mean ± standard deviation (SD) or median where appropriate. The sample sizes for each specific assay (*n*) are detailed in the respective figure legends and tables.

## 5. Conclusions

This study demonstrates that while clove, tea tree, and plai oils exhibit measurable antimicrobial activity, an ethanolic *P. betle* leaf extract provides superior broad-spectrum efficacy against *P. aeruginosa* and *Staphylococcus* spp. clinical isolates from dogs and cats. Notably, despite the high prevalence of strong biofilm production among these isolates, the *P. betle* extract consistently exhibited the most potent antibiofilm activity, as reflected by its lower MBIC values. The GC–MS and LC–MS/MS-QTOF profiles suggest that a combination of phytochemicals likely drives this enhanced performance. Volatile constituents such as chavibetol may contribute to antibacterial activity, while phenolic compounds—including hydroxychavicol, 2,3-dihydroxybenzoic acid, and 4-hydroxybenzoic acid—likely contribute to the extract’s multi-targeted efficacy, including potential iron-chelating and antibiofilm effects. Although clove oil demonstrated strong activity against *Staphylococcus* spp., the broader and more consistent performance of the *P. betle* extract across both Gram-positive and Gram-negative pathogens highlights its distinct therapeutic potential. Collectively, these findings identify *P. betle* leaf extract as a promising plant-based candidate for topical antiseptic formulations. Such applications may be particularly valuable in managing multidrug-resistant, biofilm-associated skin and ear infections in veterinary medicine, offering a rational, evidence-based complementary strategy to address the global challenge of antimicrobial resistance.

## Figures and Tables

**Table 1:** Inhibition zones of essential oils, *Piper betle* leaf extract, and gentamicin against *Pseudomonas aeruginosa* ATCC 27853 and *Staphylococcus aureus* ATCC 6538 using a disk diffusion assay.

Plant Extract/Antibiotic	Diameter of Inhibition Zone (mm)	
	*P*. *aeruginosa* ATCC 27853	*S*. *aureus* ATCC 6538
Cinnamon oil	11.2 ± 0.4	15.0
Clove oil	10.5 ± 0.7	16.0 ± 0.7
Plai oil	7.0	13.5 ± 0.7
Tea tree oil	6.7 ± 0.4	18.0
*P. betle* leaf oil	6.6	16.0 ± 1.4
Lemongrass oil	6.5	24.7 ± 1.8
Citronella oil	No zone of inhibition	14.5 ± 0.7
Galanga oil	No zone of inhibition	8.0 ± 0.7
Turmeric oil	No zone of inhibition	7.0
*P. betle* leaf extract (32 mg/mL)	22.5 ± 3.5	22.8 ± 0.7
Gentamicin disk (10 µg)	20.5 ± 0.7	26.5 ± 0.5

Values represent the mean ± standard deviation (SD) of three independent replicates. Values reported without an SD indicate that identical results were obtained across all replicates.

**Table 2:** Minimum inhibitory concentrations (MICs) of essential oils and *Piper betle* leaf extract screening against reference and clinical strains of *Pseudomonas aeruginosa* and *Staphylococcus* spp.

Essential Oils/ Ethanolic Extract	MICs to Bacterial Strains (mg/mL)			
	*P. aeruginosa* ATCC 27853	*P. aeruginosa* Strains (*n* = 5) *	*S. aureus* ATCC 6538	*Staphylococcus* Strains (*n* = 4) *
Cinnamon oil	8.0	48.5 ± 24.5 ^b,1^	0.3 ± 0.1	0.5 ± 0.4 ^2^
Clove oil	8.0	5.3 ± 2.8 ^a,1^	0.8 ± 0.3	0.7 ± 0.2 ^2^
Plai oil	6.7 ± 2.3	10.4 ± 6.4 ^a,1^	2.7 ± 1.2	4.0 ± 2.9 ^2^
Tea tree oil	4.0	8.0 ± 4.7 ^a,1^	1.3 ± 0.6	3.6 ± 3.2 ^2^
*P. betle* leaf oil	64	43.5 ± 28.8 ^b,1^	1.0	0.8 ± 0.8 ^2^
Lemongrass oil	10.7 ± 4.6	22.4 ± 9.5 ^b,1^	0.3 ± 0.1	0.3 ± 0.1 ^2^
*P. betle* leaf extract	1.0	1.2 ± 0.5 ^a,1^	0.3 ± 0.1	0.2 ± 0.1 ^2^

The MICs of cinnamon against *P. aeruginosa* strains were determined to be 64 mg/mL and were used directly in calculations, rather than being expressed as >64 mg/mL. ^a,b^ Mean values within the same column carrying different superscripts are significantly different based on the Kruskal–Wallis One-Way ANOVA (*p* < 0.05); the significance threshold for pairwise comparisons was adjusted to *p* < 0.0024 using the Bonferroni correction, ^1,2^ Mean values within the same row carrying different superscripts are significantly different by Mann–Whitney U test (*p* < 0.05), and * clinical isolates. Test results without a standard deviation (SD) indicate that identical values were obtained across all three replicates.

**Table 3:** Minimum inhibitory concentration (MIC_50_ and MIC_90_) of plant extracts against clinical isolates of *Pseudomonas aeruginosa* and *Staphylococcus* spp.

Plant Extract	*P. aeruginosa* (*n* = 43)		*Staphylococcus* spp. (*n* = 30)	
	MIC_50_ (mg/mL)	MIC_90_ (mg/mL)	MIC_50_ (mg/mL)	MIC_90_ (mg/mL)
*P. betle* leaf extract	1.0	2.0	ND	ND
Clove oil	4.0	4.0	1.0	1.0
Tea tree oil	8.0	8.0	2.0	4.0
Plai oil	16.0	32.0	1.0	4.0

ND: not determined.

**Table 4:** Gentamicin and multidrug resistance rates in *Pseudomonas aeruginosa* from dogs and cats and in canine *Staphylococcus* isolates.

Antimicrobial Resistance *	*P. aeruginosa* (*n* = 43)		*Staphylococcus* spp. (Canine, *n* = 30)		
	Dogs (*n* = 29)	Cats (*n* = 14)	MRSP (*n* = 10)	MSSP (*n* = 10)	MRSS (*n* = 10)
Gentamicin	5 (17.2%)	2 (14.2%)	6 (60.0%)	2 (20.0%)	2 (20.0%)
MDR	5 (17.2%)	2 (14.2%)	8 (80.0%)	0 (0.0%)	7 (70.0%)

* resistance included both resistant and intermediate isolates according to the interpretive criteria. MDR, multidrug resistance; MRSP, methicillin-resistant *Staphylococcus pseudintermedius*; MSSP, methicillin-susceptible *Staphylococcus pseudintermedius*; and MRSS, methicillin-resistant *Staphylococcus schleiferi*.

**Table 5:** Biofilm-forming ability of clinical *Pseudomonas aeruginosa* and *Staphylococcus* spp.

Biofilm Formation	*P. aeruginosa* (*n* = 43)			*Staphylococcus* spp. (*n* = 30)		
	MDR (*n* = 7)	Non-MDR (*n* = 36)	Total (*n* = 43)	MRSP (*n* =10)	MSSP (*n* = 10)	MRSS (*n* = 10)
Strong	6 (85.7%)	26 (72.2%)	32 (74.4%)	10 (100%)	9 (90%)	8 (80%)
Moderate	1 (14.3%)	10 (27.8%)	11 (25.6%)	0 (0%)	1 (10%)	2 (20%)

MDR, multidrug resistance; MRSP, methicillin-resistant *Staphylococcus pseudintermedius*; MSSP, methicillin-susceptible *Staphylococcus pseudintermedius*; and MRSS, methicillin-resistant *Staphylococcus schleiferi*.

**Table 6:** Minimal biofilm inhibitory concentration (MBIC) and biofilm inhibitory rate of gentamicin against clinical *Pseudomonas aeruginosa* and *Staphylococcus* spp.

Gentamicin Susceptibility	Gentamicin MIC (mg/mL) *	Gentamicin MBIC (mg/mL)	Biofilm Inhibitory Rate at 0.002 mg/mL (%)	Biofilm Inhibitory Rate at 0.004 mg/mL (%)	Biofilm Inhibitory Rate at 0.008 mg/mL (%)	Biofilm Inhibitory Rate at 0.016 mg/mL (%)
*P. aeruginosa*						
Susceptible (*n* = 4)	≤0.0010	0.0017	101.4 ± 3.5	102.3 ± 2.7	102.9 ± 3.1	102.7 ± 3.3
Resistant (*n* = 1)	0.0080	0.0160	37.9 ± 0.5	67.9 ± 0.1	87.5 ± 0.1	100 ± 0.1
Resistant (*n* = 2)	≥0.0160	>0.0160	33.8 ± 6.6	36.1 ± 2.3	46.9 ± 1.7	50.2 ± 1.6
*Staphylococcus* spp.						
Susceptible MSSP (*n* = 1)	≤0.0005	>0.0160	22.9 ± 0.1	45.5 ± 0.1	82.6 ± 0.1	85.1 ± 0.1
Susceptible MRSP (*n* = 1)	≤0.0005	0.0002	99.2 ± 0.1	99.4 ± 0.1	99.8 ± 0.1	100.8 ± 0.1
Susceptible MRSS (*n* = 3)	≤0.0005	0.0070	62.2 ± 30.3	89.6 ± 0.6	98.1 ± 0.7	96.6 ± 7.3
Resistant MRSP (*n* = 3)	≥0.0160	>0.0160	25.3 ± 9.1	27.3 ± 13.2	44.6 ± 19.1	51.5 ± 15.3
Resistant MRSS (*n* = 2)	≥0.0160	>0.0160	11.9 ± 11.9	15.1 ± 19.3	46.1 ± 48.2	52.2 ± 41.4

* Gentamicin MIC for *P. aeruginosa* and *Staphylococcus* spp. was determined using the VITEK2 Compact system. MRSP, methicillin-resistant *Staphylococcus pseudintermedius*; MSSP, methicillin-susceptible *Staphylococcus pseudintermedius*; MRSS, methicillin-resistant *Staphylococcus schleiferi*; and MIC, minimum inhibitory concentration. Values for biofilm inhibitory rates represent the mean ± SD of *n* isolates, with each isolate tested in triplicate. For groups where *n* = 1, values represent the mean of three independent replicates. The high standard deviation observed is attributed to the limited sample size (*n* = 2) and inherent inter-isolate variability.

**Table 7:** The chemical composition of the plant extracts—clove oil, tea tree oil, plai oil, lemongrass oil, *Piper betle* leaf oil, and *P. betle* leaf extract—was characterized by GC-MS.

Group	Compound	Retention Index	CO		TTO		PO		LMO		BTO		BTX	
			%A	T_R_	%A	T_R_	%A	T_R_	%A	T_R_	%A	T_R_	%A	T_R_
Phenyl- propanoids	Chavicol acetate *	1315 *	-	-	-	-	-	-	-	-	3.1	11.3	7.0	9.9
	Chavibetol	1359	-	-	-	-	-	-	-	-	19.8	11.7	44.2	11.7
	Eugenol	1359	83.4	22.7	-	-	-	-	-	-	-	-	-	-
	Eugenol acetate	1522	-	-	-	-	-	-	-	-	14.2	13.7	-	-
	Hydroxychavicol	1424	-	-	-	-	-	-	-	-	-	-	26.3	12.9
	4-allyl-1,2-diacetoxybenzene	-	-	-	-	-	-	-	-	-	6.2	15.2	-	-
Sesquiter- penes	γ-Muurolene	1454	-	-	-	-	-	-	-	-	10.3	13.2	5.3	13.2
	α-Selinene	1489	-	-	-	-	-	-	-	-	-	-	0.7	13.6
	Trans- caryophyllene	1419	10.5	24.9	0.5	12.5	-	-	-	-	7.5	12.5	-	-
	δ-Cadinene	1523	-	-	2.2	13.7	-	-	-	-	7.2	13.7	2.1	13.7
	cis-Calamenene	1528	-	-	-	-	-	-	-	-	-	-	1.3	13.8
	α -Calacorene	1544	-	-	-	-	-	-	-	-	-	-	0.5	14.1
	Spathulenol	1578	-	-	-	-	-	-	-	-	-	-	0.6	14.6
	Globulol	1590	-	-	0.5	14.7	-	-	-	-	1.3	14.7	0.4	14.7
	Ledene	1496	-	-	-	-	-	-	-	-	4.1	13.5	-	-
	Caryophyllene oxide	1583	2.7	30.1	-	-	-	-	-	-	-	-	-	-
	α-Humulene	1454	2.6	26.1	-	-	-	-	-	-	-	-	-	-
Terpineol	Terpinen-4-ol	1177	-	-	47.9	9.1	64.8	28.9	0.6	8.9	-	-	-	-
	γ-Terpinene	1059	-	-	8.4	6.7	-	-	-	-	-	-	-	-
	Geranial	1267	-	-	-	-	-	-	48.2	10.2	-	-	-	-
	Neral	1238	-	-	-	-	-	-	33.7	9.7	-	-	-	-
	α-Tepineol	1188	-	-	6.3	9.1	3.7	29.9	-	-	-	-	-	-
	Sabinene	975	-	-	-	-	5.5	11.5	-	-	-	-	-	-
	*p*-Cymene	1024	-	-	8.9	6.1	4.5	14.8	-	-	-	-	-	-
Others			0.8		25.3		21.5		17.5		26.5		11.6	

%A, % peak area; T_R_, retention time; CO, clove oil; TTO, tea tree oil; PO, plai oil; LMO, lemongrass oil; BTO, *Piper betle* leaf oil; BTX, *P. betle* leaf extract; Dash (-), not detected. * [44].

**Table 8:** Chemical constituents of the *Piper betle* leaf extract identified via LC-MS/MS-QTOF.

Analyte Peak Name	Retention Time (min)	% Peak Area	Formula	Precursor Mass	Found at Mass	Library Score
Ornithine	1.0	1.4	C_5_H_12_N_2_O_2_	130.889	130.889	100
D-Arabinonic acid	1.0	4.1	C_5_H_10_O_6_	164.837	164.837	91.1
D- (+)-Mannose	1.2	7.7	C_6_H_12_O_6_	179.056	179.056	91.1
Uridine	1.1	1.9	C_9_H_12_N_2_O_6_	279.039	279.040	98.4
4-O-β-Galactopyranosyl-D-mannopyranose	1.1	10.1	C_12_H_22_O_11_	377.086	377.086	97.5
D- (+)-Trehalose	1.2	6.2	C_12_H_22_O_11_	341.109	341.109	100
Pyrocatechol	2.8	14.1	C_6_H_6_O_2_	109.029	109.029	97.2
2,3-Dihydroxybenzoic acid	2.8	20.2	C_7_H_6_O_4_	153.019	153.019	94.3
4-Hydroxybenzoic acid	3.6	4.2	C_7_H_6_O_3_	137.024	137.023	97.5
3-Hydroxybenzyl alcohol	3.7	14.9	C_7_H_8_O_2_	123.044	123.044	100
3,4-Dihydroxybenzaldehyde	8.7	15.2	C_7_H_6_O_3_	137.023	137.023	97.3

**Table 9:** Comparative antimicrobial concentrations reported for topical veterinary antiseptics and plant-derived agents against *Pseudomonas aeruginosa* and *Staphylococcus* spp.

Agent	Typical MIC or Active Concentration	Clinical Context	Reference Type
Gentamicin	0.5–2 µg/mL	Topical antibiotic commonly used in veterinary otitis and pyoderma	Experimental/QC
Chlorhexidine	0.5–4% formulations	Standard veterinary antiseptic shampoo and rinse	[27]
Silver sulfadiazine	0.001–0.064 mg/mL	Burn wounds and resistant infections	[54]
Tris-EDTA combinations	Tris: 2.2–8.9 mg/mL EDTA: 0.6–2.4 mg/mL	Veterinary otitis management	[29]
*P. betle* leaf extract	0.7–2 mg/mL	Experimental antibiofilm and antimicrobial activity	Present study
Clove oil	0.8–4 mg/mL	Plant-derived topical candidate	Present study

QC, quality control.

**Table 10:** The analytical parameters used are specific to each essential oil.

Plant Extracts	Injector Temperatures	Transfer Line Temperature	Oven Temperature Programmed	Split Ratio	Scan Mode
*P. betle* leaf oil Tea tree oil Lemongrass oil	280 °C	250 °C	80 °C for 3 min raise up 10 °C/min until 250 °C for 4 min	1:20	35–500
Clove oil	230 °C	220 °C	60 °C for 3 min raise up 4 °C/min until 220 °C for 10 min	1:10	40–400
Plai oil	250 °C	250 °C	60 °C for 3 min raise up 1 °C/min until 80 °C raise up 3 °C/min until 120 °C raise up 4 °C/min until 220 °C for 10 min	1:7	40–350

## Data Availability

All data generated or analyzed during this study are included in this manuscript and its Supplementary Material Files.

## Supplementary Materials

The following supporting information can be downloaded at: https://www.mdpi.com/article/10.3390/antibiotics15060549/s1, Table S1: Multiple pairwise comparisons of the minimum inhibitory concentrations (MICs) for essential oils and *Piper betle* leaf extract against clinical strains of *Pseudomonas aeruginosa* (*n* = 5), with significance determined by Bonferroni correction; Table S2: Multiple pairwise comparisons of the minimum inhibitory concentrations (MICs) and minimum bactericidal concentrations (MBCs) for *Piper betle* leaf extract, clove oil, tea tree oil, and plai oil against clinical *Pseudomonas aeruginosa* and *Staphylococcus* strains, with significance determined by Bonferroni correction; Table S3: Multiple pairwise comparisons of the minimum biofilm inhibitory concentration (MBIC) values for plant extracts against clinical *Pseudomonas aeruginosa* and *Staphylococcus* strains, with significance determined by Bonferroni correction; Table S4: Multiple pairwise comparisons of antibiofilm activity for *Piper betle* leaf extract, clove oil, tea tree oil, and plai oil at different concentrations (0.5 MBIC, MBIC, and 2 MBIC) against clinical *Pseudomonas aeruginosa* and *Staphylococcus* strains, with significance determined by Bonferroni correction; Table S5: Chemical composition of clove oil characterized by GC-MS; Table S6: Chemical composition of tea tree oil characterized by GC-MS; Table S7: Chemical composition of plai oil characterized by GC-MS; Table S8: Chemical composition of lemongrass oil characterized by GC-MS; Table S9: Chemical composition of *Piper betle* leaf oil characterized by GC-MS; Table S10: Chemical composition of *Piper betle* leaf extract characterized by GC-MS; Table S11: Published safety data supporting topical feasibility of *Piper betle*-derived compounds; Table S12: Physical properties of *Piper betle* leaf extract (BTX) and essential oils, based on the manufacturers’ certificates of analysis and the authors’ direct observations of the BTX extract.
