# Supplementary material for: Phytochemical Characterization and Antibiofilm Efficacy of Piper betle Extract and Essential Oils Against Clinical Pseudomonas aeruginosa and Staphylococcus spp. from Small Animals
Source: Antibiotics (Basel). 2026 May 29;15(6):549. doi: 10.3390/antibiotics15060549 (PMC13295859; doi:10.3390/antibiotics15060549)
Supplement: Supplementary file 1 [file antibiotics-15-00549-s001.zip › antibiotics-4270173-supplementary.pdf]

Supplementary Table S1. Multiple pairwise comparisons of the minimum inhibitory concentrations (MICs) for essential oils and *Piber betle* leaf extract against clinical strains of *Pseudomonas aeruginosa* (n = 5), with significance determined by Bonferroni correction.

Comparison pair	Statistical significance	*p-value*
BTX vs. CO	No	0.9993
BTX vs. TTO	No	0.9916
BTX vs. PO	No	0.9672
BTX vs. LMO	Yes	0.0007
BTX vs. BTO	Yes	0.0013
BTX vs. CNO	Yes	0.0006
BTO vs. CO	Yes	0.0010
BTO vs. TTO	Yes	0.0015
BTO vs. PO	Yes	0.0018
BTO vs. LMO	No	0.3062
BTO vs. CNO	No	0.9989
CO vs. TTO	No	1.0000
CO vs. PO	No	0.9990
CO vs. LMO	Yes	0.0011
CO vs. CNO	Yes	0.0007
TTO vs. PO	No	1.0000
TTO vs. LMO	Yes	0.0013
TTO vs. CNO	Yes	0.0004
PO vs. LMO	Yes	0.0016
PO vs. CNO	Yes	0.0009
LMO vs. CNO	No	0.3293

BTX, *P. betle* leaf extract; CO, clove oil; TTO, tea tree oil; PO, plai oil; LMO, lemongrass oil; BTO, *P. betle* leaf oil; CNO, cinnamon oil. Significance was established at $p < 0.0024$ (Bonferroni-adjusted $\alpha = 0.05/21$ comparisons).

Supplementary Table S2. Multiple pairwise comparisons of the minimum inhibitory concentrations (MICs) and minimum bactericidal concentrations (MBCs) for *Piper betle* leaf extract, clove oil, tea tree oil, and plai oil against clinical *Pseudomonas aeruginosa* and *Staphylococcus* strains, with significance determined by Bonferroni correction.

MIC			MBC		
**Comparison pair**	**Statistical significance**	***p*-value**	**Comparison pair**	**Statistical significance**	***p*-value**
		P. aeruginosa (*n* = 43)			
BTX vs. CO	Yes	0.0041	BTX vs. CO	Yes	<0.0001
BTX vs. TTO	Yes	<0.0001	BTX vs. TTO	Yes	<0.0001
BTX vs. PO	Yes	<0.0001	BTX vs. PO	Yes	<0.0001
CO vs. TTO	Yes	<0.0001	CO vs. TTO	Yes	0.0015
CO vs. PO	Yes	<0.0001	CO vs. PO	Yes	<0.0001
TTO vs. PO	Yes	0.0064	TTO vs. PO	Yes	0.0071
		Staphylococcus spp. (*n* = 30)			
CO vs. TTO	Yes	<0.0001	CO vs. TTO	Yes	<0.0001
CO vs. PO	Yes	0.0009	CO vs. PO	Yes	0.0016
TTO vs. PO	No	0.5197	TTO vs. PO	No	0.2758

BTX, *P. betle* leaf extract; CO, clove oil; TTO, tea tree oil; PO, plai oil. Statistical significance was established using Bonferroni-corrected alpha levels: $p < 0.008$ for *P. aeruginosa* (6 comparisons) and $p < 0.017$ for *Staphylococcus* spp. (3 comparisons).

Supplementary Table S3. Multiple pairwise comparisons of the minimum biofilm inhibitory concentration (MBIC) values for plant extracts against clinical *Pseudomonas aeruginosa* and *Staphylococcus* strains, with significance determined by Bonferroni correction.

Comparison pair	Statistically significance	*p*-value
	Pseudomonas aeruginosa (n = 43)	
BTX vs. CO	Yes	<0.0001
BTX vs. TTO	Yes	<0.0001
CO vs. TTO	Yes	0.0001
	Staphylococcus spp. (n = 30)	
BTX vs. CO	No	0.0132
BTX vs. TTO	Yes	<0.0001
BTX vs. PO	Yes	<0.0001
CO vs. TTO	Yes	<0.0001
CO vs. PO	Yes	0.0014
TTO vs. PO	No	>0.9999

BTX, *P. betle* leaf extract; CO, clove oil; TTO, tea tree oil; PO, plai oil. Statistical significance was established using Bonferroni-corrected alpha levels: $p < 0.017$ for *P. aeruginosa* (3 comparisons) and $p < 0.008$ for *Staphylococcus* spp. (6 comparisons).

Supplementary Table S4. Multiple pairwise comparisons of antibiofilm activity for *Piper betle* leaf extract, clove oil, tea tree oil, and plai oil at different concentrations (0.5 MBIC, MBIC, and 2 MBIC) against clinical *Pseudomonas aeruginosa* and *Staphylococcus* strains, with significance determined by Bonferroni correction.

Comparison pair	Statistically significance	*p*-value
	P. aeruginosa (*n* = 43)	
BTX0.5MBIC vs. BTXMBIC	Yes	<0.0001
BTX0.5MBIC vs. BTX2MBIC	Yes	<0.0001
BTXMBIC vs. BTX2MBIC	No	0.85992
CO0.5MBIC vs. COMBIC	Yes	<0.0001
CO0.5MBIC vs. CO2MBIC	Yes	<0.0001
COMBIC vs. CO2MBIC	No	0.45921
TTO0.5MBIC vs. TTOMBIC	Yes	<0.0001
TTO0.5MBIC vs. TTO2MBIC	Yes	<0.0001
TTOMBIC vs. TTO2MBIC	No	0.64924
	Staphylococcus spp. (*n* = 30)	
BTX0.5MBIC vs. BTXMBIC	Yes	<0.0001
BTX0.5MBIC vs. BTX2MBIC	Yes	<0.0001
BTXMBIC vs. BTX2MBIC	No	0.85571
CO0.5MBIC vs. COMBIC	Yes	<0.0001
CO0.5MBIC vs. CO2MBIC	Yes	<0.0001
COMBIC vs. CO2MBIC	No	0.91773
TTO0.5MBIC vs. TTOMBIC	Yes	<0.0001
TTO0.5MBIC vs. TTO2MBIC	Yes	<0.0001
TTOMBIC vs. TTO2MBIC	No	0.8829
PO0.5MBIC vs. POMBIC	Yes	<0.0001
PO0.5MBIC vs. PO2MBIC	Yes	<0.0001
POMBIC vs. PO2MBIC	No	0.95572

BTX, *P. betle* leaf extract; CO, clove oil; TTO, tea tree oil; PO, plai oil; MBIC, minimum biofilm inhibitory concentration. Significance was established at $p < 0.017$ (Bonferroni-adjusted $\alpha = 0.05/3$ comparisons within each extract-strain group).

Supplementary Table S5. Chemical composition of clove oil characterized by GC-MS.

No	Retention time	% Peak area	Retention index	Compounds
1	22.689	83.37	1359	Eugenol
2	24.962	10.53	1419	trans-Caryophyllene
3	26.119	2.6	1454	α-Humulene
4	28.029	0.29	1523	γ-Cadinene
5	30.067	2.76	1583	Caryophyllene oxide
6	30.892	0.44	1608	Humulene epoxide II

Supplementary Table S6. Chemical composition of tea tree oil characterized by GC-MS.

No	Retention time	% Peak area	Retention index	Compounds
1	4.372	0.73	930	α-Thujene
2	4.543	2.58	939	α-Pinene
3	5.348	0.75	979	β-Pinene
4	5.993	2.42	1017	α-Terpinene
5	6.129	8.92	1024	*p*-Cymene
6	6.217	0.61	1029	D-Limonene
7	6.270	0.42	1029	β-Phellandrene
8	6.319	3.78	1031	Eucalyptol
9	6.718	8.36	1059	γ-Terpinene
10	7.211	1.55	1088	α-Terpinolene
11	8.219	0.35	1140	trans-p-Menth-2-en-1-ol
12	8.885	47.9	1177	Terpinen-4-ol
13	9.093	6.33	1188	α-Terpineol
14	10.352	1.00	1270	1,4-Dihydroxy-p-menth-2-ene
15	10.608	0.80	-	unidentified
16	11.036	0.83	-	unidentified
17	11.663	0.36	-	unidentified
18	12.333	0.59	1409	α-Gurjunene
19	12.527	0.47	1419	trans-Caryophyllene
20	12.779	2.26	1441	Aromadendrene
21	12.867	0.47	1454	Selina-5,11-diene
22	13.073	0.94	1460	Alloaromadendrene
23	13.175	0.54	1472	β-Cadinene
24	13.383	0.57	1486	p-Menthane-1,2,4-triol
25	13.449	2.08	1496	Viridiflorene
26	13.743	2.23	1523	δ-Cadinene
27	13.817	0.88	1528	cis-Calamenene
28	13.954	0.32	1538	α-Cadinene
29	14.700	0.48	1590	Globulol
30	14.817	0.49	1592	Viridiflorol

Supplementary Table S7. Chemical composition of plai oil characterized by GC-MS.

No	Retention time	% Peak area	Retention index	Compound
1	8.354	0.55	930	α-Thujene
2	8.748	1.14	939	α-Pinene
3	11.047	5.52	975	Sabinene
4	11.352	1.94	979	α-Pinene
5	14.778	4.45	1024	p-Cymene
6	24.125	1.14	1070	cis-Sabinene hydrate
7	25.784	0.95	1098	trans-Sabinene hydrate
8	28.875	64.83	1177	Terpinen-4-ol
9	29.845	2.91	-	unidentified
10	29.921	3.74	1188	α-terpineol
11	33.747	0.53	-	unidentified
12	35.015	0.88	-	trans-p-Menth-2-ene-1,4-diol
13	36.056	0.66	1276	cis-Verbenol acetate
14	37.648	0.55	-	unidentified
15	39.596	0.82	-	unidentified
16	44.689	0.99	1486	(1S,2S,4S)-Trihydroxy-p-menthane
17	45.894	0.91	1521	β-Sesquiphellandrene
18	48.096	3.46	1616*	(E)-1-(3,4-Dimethoxyphenyl) but-1-ene
19	49.269	3.35	1662*	(E)-1-(3',4'-Dimethoxyphenyl) butadiene
20	53.542	0.67	1720**	3-(3,4-dimethoxyphenyl)-2-propenal

*National Center for Biotechnology Information (NCBI), National center library of medicine, PubChem

**National Institute of Standards and Technology (NIST), NIST Chemistry WebBook

Supplementary Table S8. Chemical composition of lemongrass oil characterized by GC-MS.

No	Retention time	% Peak area	Retention index	Compound
1	5.332	1.34	974	6-Methylhept-5-en-2-one
2	7.406	1.25	1096	Linalool
3	8.89	0.61	1177	Terpinen-4-ol
4	9.491	0.37	1225	Citronellol
5	9.733	33.68	1238	Neral
6	9.871	3.8	1252	Geraniol
7	10.069	0.65	1252	Piperitone
8	10.174	48.17	1267	Geranial
9	10.403	0.6	-	Epoxy-linalooloxide
10	10.488	0.45	-	Unknown compound base peak at m/z=82
11	10.597	46	1282	Neryl formate
12	10.775	0.78	-	Unknown compound base peak at m/z=126
13	11.254	1.01	-	trans-Geranoic acid
14	11.69	0.76	-	Unknown compound base peak at m/z=82
15	11.729	2.54	-	Geranyl acetate
16	12.21	0.41	-	Unidentified
17	12.617	0.45	-	α-trans-Bergamotene
18	13.854	0.38	-	Isoascaridol
19	14.694	0.37	1583	Caryophyllene oxide
20	15.163	0.93	1624	Selina-6-en-4-ol

Supplementary Table S9. Chemical composition of *Piper betle* leaf oil characterized by GC-MS.

No.	Retention time	% Peak area	Retention index	Compound
1	6.314	1.16	1031	Eucalyptol
2	7.393	0.65	1096	Linalool
3	9.864	0.69	1250	Chavicol
4	11.253	3.05	1315*	Chavicol acetate
5	11.673	19.82	1359	Chavibetol
6	11.867	2.04	1376	Copaene
7	12.017	2.63	1390	β-Elemene
8	12.328	0.38	1412	Cis-α-Bergamotene
9	12.525	7.45	1419	trans- Caryophyllene
10	12.645	0.7	1433	β-Gurjunene
11	12.772	1.69	1441	Aromdendrene
12	13.010	2	1454	α-Humulene
13	13.202	10.3	1479	γ-Muurolene
14	13.26	0.72	1484	α-Amorphene
15	13.331	1.25	1485	Germacrene D
16	13.447	4.05	1496	Ledene
17	13.496	1.98	1500	α-Muurolene
18	13.535	1.44	1522	7-epi-α-Selinene
19	13.638	14.15	1522	Eugenol acetate
20	13.742	7.22	1523	δ-Cadinene
21	13.813	1.59	1528	Cis-Calamenene
22	14.569	0.75	1578	Spathulenol
23	14.697	1.29	1590	Globulol
24	14.814	1.07	1592	Viridiflorol
25	14.943	0.38	1600	Rosifoliol
26	15.022	6.17	-	4-Allyl-1,2-diacetoxybenzene
27	15.165	1.05	1628	epi-Cubenol
28	15.34	1.96	1640	epi-α-Cadinol
29	15.482	1.75	1654	α-Cadinol
30	15.569	0.62	1659	Selin-11-en-4-α-ol

*The retention index value is based on reference [44].

Supplementary Table S10. Chemical composition of *Piper betle* leaf extract characterized by GC-MS.

No.	Retention time	% Peak	Retention index	Compound
1	6.570	0.61	931	3-Ethyl-3-methylheptane
2	9.875	7.02	1195	Chavicol, acetate
3	10.23	0.63	1229	5-Methyl-5-propylnonane
4	11.675	44.17	1392	Chavibetol
5	12.550	0.70	1417	Trans-Caryophyllene
6	12.970	26.34	1424	Hydroxychavicol
7	13.220	5.27	1478	γ-Muurolene
8	13.475	1.84	-	2,4-Di-tert-butyl phenol
9	13.575	0.69	1489	α-Selinene
10	13.740	2.11	1523	δ-Cadinene
11	13.840	1.33	1528	Cis-Calamenene
12	14.105	0.55	1544	α -Calacorene
13	14.610	0.58	1578	Spathulenol
14	14.670	0.44	1590	Globulol
15	15.835	0.51	1612	Hexadecane
16	16.335	0.95	1701	n-Pentadecanal
17	17.005	0.62	1774	Tua-Cadinol acetate
18	17.360	0.50	1774	Neophytadiene
19	18.605	1.55	1968	n-Hexadecanoic acid
20	18.965	3.6	1978	Hexadecanoic acid, ethyl ester

Supplementary Table S11. Published safety data supporting topical feasibility of *Piper betle*-derived compounds.

Compound/Extract	Experimental model	Reported non-toxic concentration	Reported effect
Piper betle extract	Keratinocyte culture	Approximately equivalent to active MIC range (1.5 mg/mL)	No significant cytotoxicity [56]
Hydroxychavicol	Fibroblast/epithelial cells	Sub-mg/mL to low mg/mL range (50-100 μg/mL)	Acceptable cellular viability [58]
Eugenol-containing oils	Topical dermatological studies	Concentration dependent	Mild irritation at higher concentrations [57]

Supplementary Table S12. Physical properties of *Piper betle* leaf extract (BTX) and essential oils, based on the manufacturers' certificates of analysis and the authors' direct observations of the BTX extract

Plant extracts	Color and appearance	Odor	Solubility	Specific gravity (20/20˚C)
Cinnamon oil	Clear, pale yellow to bright yellow to orange to red, amber to dark brown	Spicy and intensely sweet aroma	Soluble in most fixed oils	1.0443
Clove oil	Colorless to yellow-brown liquid. Long time, color change deeper	Spicy-sweet, green, sweet, green-rich clove floral odor	NA	NA
Plai oil	Colorless to yellow and clear liquid	Special plai characteristic herbaceous odor	Slightly soluble in ethanol	0.8800-0.9250
Tea tree oil	Colorless to pale yellow and clear liquid	Cool green herbal odor	NA	NA
P. betle leaf oil	Yellow to yellow-brown and clear liquid	Green, spicy, herby odor	Soluble in 1.5 parts of 80% ethyl alcohol	1.0132-1.0532
Lemongrass oil	Yellow, yellow-brown or reddish brown, and clear liquid	Fresh green sweet lemon fruit odor	Practically insoluble in ethanol, soluble in organic solvents	0.8750-0.8850
Citronella oil	Pale yellow to yellow and clear liquid	Sweet and green grass odor	Soluble in 2 parts of 80% ethyl alcohol	0.8780-0.9140
Galanga oil	Colorless to lemon-yellow and clear liquid	Fresh, spicy-camphoraceous odor and herby note	Practically insoluble in ethanol, soluble in organic solvents	0.8950-0.9150
Turmeric oil	Yellow to yellow-lemon and clear liquid	Special herby note characteristic, spicy turmeric odor	Soluble in 0.5 parts of 90% ethyl alcohol. Also soluble in oils and organic solvents	0.9160-0.9366
P. betle leaf extract	Dense, syrupy, dark greenish-brown to near-black extract.	Special herb note characteristic, fresh green *P. betle* leaf	Soluble in ethanol and DMSO	NA
